# The Role of the Metzincin Superfamily in Prostate Cancer Progression: A Systematic-Like Review

**DOI:** 10.3390/ijms22073608

**Published:** 2021-03-30

**Authors:** Marley J. Binder, Alister C. Ward

**Affiliations:** School of Medicine, Deakin University, Geelong, VIC 3216, Australia; m.binder@deakin.edu.au

**Keywords:** metzincin, prostate cancer, Matrix Metalloproteinase, Tissue Inhibitor of Metalloproteinases, A Disintegrin and Metalloproteinase, A Disintegrin and Metalloproteinase with Thrombospondin Motifs

## Abstract

Prostate cancer remains a leading cause of cancer-related morbidity in men. Potentially important regulators of prostate cancer progression are members of the metzincin superfamily of proteases, principally through their regulation of the extracellular matrix. It is therefore timely to review the role of the metzincin superfamily in prostate cancer and its progression to better understand their involvement in this disease. A systematic-like search strategy was conducted. Articles that investigated the roles of members of the metzincin superfamily and their key regulators in prostate cancer were included. The extracted articles were synthesized and data presented in tabular and narrative forms. Two hundred and five studies met the inclusion criteria. Of these, 138 investigated the role of the Matrix Metalloproteinase (MMP) subgroup, 34 the Membrane-Tethered Matrix Metalloproteinase (MT-MMP) subgroup, 22 the A Disintegrin and Metalloproteinase (ADAM) subgroup, 8 the A Disintegrin and Metalloproteinase with Thrombospondin Motifs (ADAMTS) subgroup and 53 the Tissue Inhibitor of Metalloproteinases (TIMP) family of regulators, noting that several studies investigated multiple family members. There was clear evidence that specific members of the metzincin superfamily are involved in prostate cancer progression, which can be either in a positive or negative manner. However, further understanding of their mechanisms of action and how they may be used as prognostic indicators or molecular targets is required.

## 1. Introduction

Prostate cancer (PrCa) is one of the major causes of cancer-related morbidity in men worldwide [1,2]. The early stages of PrCa are androgen-dependent, but during PrCa progression, the tumors become independent of androgens [1,3]. The detection of PrCa is difficult, with symptoms often not being apparent until metastasis has occurred [1]. The use of the Prostate-Specific Antigen (PSA) test is considered a gold standard, yet remains flawed, with a considerable false-positive rate [1,4]. The survival rates for men diagnosed with PrCa have increased, although the treatment options can have significant side effects [1,2]. An increased understanding of the etiology of this disease provides the potential to develop more specific detection methods and/or alternative treatment modalities.

**Figure 1:**
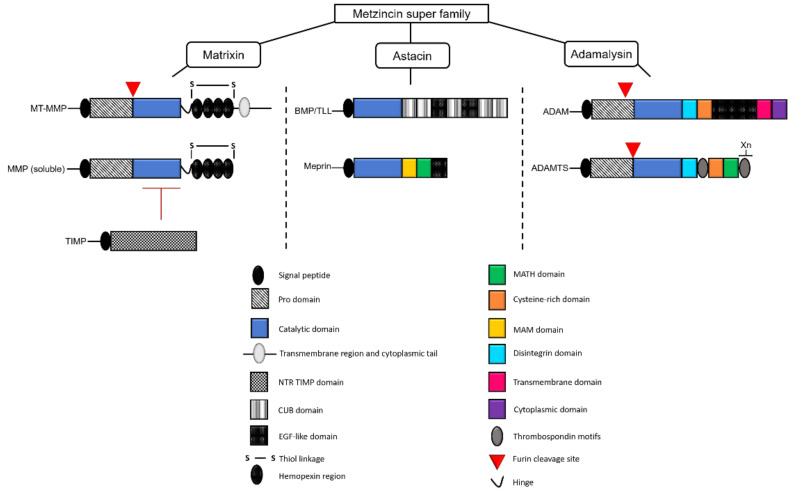
Structure of the metzincin superfamily members found in humans and their key regulators. Schematic representation of the structural components of the metzincin subgroups found in humans, grouped by family, along with the Tissue Inhibitor of Metalloproteinases (TIMP) family of regulators.

The metzincin superfamily represents a large group of proteases named after a specialized structural component, a zinc ion-binding methionine turn sequence within their catalytic domain [5,6,7]. The superfamily can be divided into families and subgroups on the basis of other structural and functional features (Figure 1). The Matrixin family consists of the soluble Matrix Metalloproteinase (MMP) and Membrane-Tethered Matrix Metalloproteinase (MT-MMP) subgroups that are principally regulated by the Tissue Inhibitor of Metalloproteinases (TIMP) family, and the Astracin family comprises the BMP1/TLL and Meprin subgroups, whereas the Adamalysin family consists of the A Disintegrin and Metalloproteinase (ADAM) and A Disintegrin and Metalloproteinase with Thrombospondin Motifs (ADAMTS) subgroups [8]. The metzincins are well-known for their roles in development and disease, largely through remodeling of the extracellular matrix (ECM) [9,10,11,12,13].

Members of the metzincin superfamily have been increasingly implicated in cancer progression, including a key role in the metastatic process via their ability to remodel the ECM of tumors [13,14,15]. However, the exact role varies, with some being tumor-promoting, others having an antitumorigenic function and others seemingly not playing a role [13,14,16,17,18]. The metzincin superfamily is therefore of interest as a potential source of biomarkers and/or targets for therapeutic interventions, although the results of the clinical trials to date have been discouraging [19]. However, given the pressing need for both biomarkers and therapeutics in PrCa, it is timely to conduct this systematic-like review in order to synthesize the role of the metzincin superfamily in this disease.

## 2. Results

Extensive database searching was undertaken to identify studies that investigated the role of metzincin superfamily members in PrCa progression, as described in Materials and Methods. This identified 205 articles that are presented in five tables, each covering a specific subgroup of the metzincin superfamily or their regulators—specifically, the Matrixin family subgroups MMPs and MT-MMPs, the TIMPs and the Adamalysin family subgroups ADAMs and ADAMTSs. A number of studies involved more than one of these groups and so are included in more than one table. No articles on the BMP/TLL or Meprin subgroups within the Astracin family were identified.

### 2.1. Soluble Matrix Metalloproteinases (MMPs)

The most extensively studied metzincin superfamily subgroup are the soluble MMPs, with 138 articles included (Table 1). Generally, MMPs have been demonstrated to act in a protumorigenic manner—particularly, MMP-2, MMP-7 and MMP-9, which have been the most widely studied of this subgroup.

The strongest evidence for protumorigenicity relates to MMP-9. Multiple publications have identified an increased expression in PrCa [59,61,78,83,109,113,129,153,156], including positive associations with more advanced PrCa [67,75,84,103] and, specifically, with higher grade/stage [26,34,53,92,138] and enhanced metastatic properties [20,21,58,65,85,146], as well as increased recurrence [92,102] and poorer prognosis [35,77,89]. Others show no association [24,47,48,52,118], and a small number show negative associations [25,31,40] with PrCa progression, indicating that MMP-9 is not universally important. However, functional studies serve to confirm its significant role, particularly in PrCa spreading, with MMP-9 ablation repeatedly shown to decrease its invasion and/or migration [57,87,100,148]. Interestingly, no differences in tumorigenesis were observed in a mouse MMP-9 knockout model [98], suggesting that expression within the tumor is important for this enzyme.

Many studies similarly showed an increased expression of MMP-2 in PrCa [49,53,59,61,62,91,150,153,156]. This also generally correlated with more advanced PrCa [32,75,84,92,94,119,132,133,141,146], including metastatic disease [54,69], as well as increased risk [47] and decreased survival [35,52,136]. In contrast, other publications showed no association [67,79,97] or a negative association [52,82,83,112,115]. In this case, a mouse MMP-2 knockout model exhibited reduced tumor burden [84], suggesting expression within the tumor is not necessarily essential for MMP-2.

Several publications have also demonstrated increased MMP-7 in PrCa [34,56,73,84,99,104,147,151], including correlations with metastasis [106] and chemoresistance [135]. However, others have identified no change in expression in PrCa [42] or, indeed, a negative correlation with disease [52], including when examining the levels of the active form of this enzyme [29,101]. For MMP-7, gene polymorphisms may be important in terms of the risk of the disease [28] and recurrence [63], while the relative levels of the inhibitors may also influence the impact of MMP-7 on PrCa progression [60]. An enforced expression of MMP-7 in PrCa cells has been shown to mediate an increased invasion [110], while a mouse MMP-7 knockout model exhibited reduced tumor-induced osteolysis [88], indicating the source of this enzyme may not be critical.

For other MMPs, there was some limited evidence that they also may play a cancer-promoting role. This includes the association of expression with PrCa for MMP-3 [64,68,79,101], MMP-10 [90,115], MMP-23 [115] and MMP-25 [115], as well as MMP-26, for which some functional evidence also exists [155].

Finally, other MMPs appear to be less significantly involved in PrCa. Thus, most studies reported no association between PrCa and MMP-1 in terms of the expression [31,48,52,68,74,83], activation [29] or polymorphism [28,44,60,63,64,68,74,81,88,90,110,155]. However, other studies have reported associations between expression and PrCa [46], including grade/stage [105,139] and metastatic properties, with MMP-1 ablation shown to reduce invasion [154]. Similarly, MMP-13 expression has typically not been associated with PrCa [31,48,84,91,95], but some studies do provide evidence of this [50,52,70]. Publications investigating MMP-11 also range from identifying no correlation [91] to a negative correlation [83] to a positive correlation [45,48], while the only study on MMP-23 points toward a negative correlation [115].

### 2.2. Membrane-Tethered Matrix Metalloproteinases (MT-MMPs)

Thirty-four articles were identified detailed the role of membrane-type MMPs in PrCa (Table 2). The majority of these related to MT1-MMP (formerly MMP-14). There were conflicting reports about whether MT1-MMP was upregulated [38,65,158] or downregulated [78,112] in PrCa, which may be partially explained by studies describing its expression as being variable across the stages of PrCa progression [67,83,101,140], with PrCa cells eliciting altered MT1-MMP expression in surrounding noncancer cells [34,36,37,151]. However, functional studies have consistently shown MT1-MMP to contribute to a more invasive/migratory phenotype [158,159,160,161,162] and, potentially, tumor growth [163,164].

A single study reported that MT2-MMP is downregulated in PrCa [38], but in contrast, MT3-MMP expression was increased and correlated with enhanced aggressiveness/metastatic potential [38,166,167,172]. Likewise, MT6-MMP expression was generally observed to be increased in PrCa [155,165], including one study that indicated a correlation with the PrCa grade [115]. The sole functional study suggested that this MT-MMP also makes a contribution to enhanced invasion [155].

### 2.3. Tissue Inhibitors of Metalloproteinases (TIMPs)

The TIMPs represent direct regulators of the metzincin superfamily—particularly, members of the MMP subgroup (Table 3). Fifty-three studies investigated the role of TIMPs in PrCa progression, which collectively indicated that these proteins typically act to suppress PrCa progression. For TIMP-1, the expression was generally reduced in PrCa [66,82,95,123,146,177], including specifically in the transition from benign to neoplastic disease [25,55,178], and was also decreased in the recurrent [113] and metastatic [179] forms of the disease. However, some studies reported increased expression in more advanced/aggressive/malignant forms [20,38,48,60,68]. This difference may in part be due to its known upregulation by inflammatory cytokines [180] that might independently impact the expression in more advanced PrCa, as well as the mode of analysis, with the protein and mRNA levels not always in correlation [82].

For TIMP-2, the included studies typically reported a reduction in expression in PrCa [55,83,112,182,188], including a negative correlation of the expression to tumor grade [105,146] and metastasis [146], with promoter hypermethylation representing one mechanism by which the expression could be lost [188]. There were also a number of conflicting studies [116,151,185,190]. However, functional investigations have demonstrated that TIMP-2 administration reduced the tumor growth [192], and enforced TIMP-2 expression reduced the tumor invasion [194].

The publications on TIMP-3 provided a similar picture, with most showing a reduced expression in PrCa [20,55,189,196], including a negative correlation with the grade [115], and with promoter hypermethylation again representing a key mechanism [191], although a couple of studies were in disagreement with this interpretation [49,52]. The functional investigations were quite definitive, however, with the ablation of TIMP-3 in mice leading to enhanced tumor growth and invasion [181] and enforced expression decreasing the proliferation, survival, migration and invasion [198], as well as increasing apoptosis and chemosensitivity [183].

Finally, there were only two studies identified on TIMP-4, one of these demonstrating an increased expression in PrCa [20] and the other one indicating a negative correlation with the grade [115].

### 2.4. A Disintegrin and Metalloproteinases (ADAMs)

Twenty-two studies investigated members of the ADAM subgroup in the context of PrCa (Table 4). A number of these provided strong evidence of positive involvement in various aspects of the disease progression. Thus, ADAM-15 expression in PrCa positively correlated with the stage, grade, metastasis and recurrence, with its ablation decreasing both the migration and metastasis [190,199,200]. ADAM-17 expression was also shown to be significantly increased in PrCa and correlated with invasiveness, with ablation decreasing the proliferation and invasiveness [201,202]. ADAM-28 expression was similarly demonstrated to be higher in PrCa, with enforced expression enhancing the proliferation and migration [203].

The results regarding ADAM-9 were more complex, with one study showing no change in expression in PrCa [189] and others showing an increased expression that correlates with malignancy and reduced survival [207,216] but another reporting a decrease in expression in castrate-resistant compared to androgen-sensitive PrCa [210]. However, the ablation of ADAM-9 reduced the proliferation and tumor growth and increased the differentiation, decreasing the metastatic ability while increasing the sensitivity to chemotherapeutic drugs [209]. For ADAM-10, the nuclear localization rather than expression was increased in PrCa, with ablation decreasing the growth [204]. For ADAM-12, the serum levels have been demonstrated to be increased in PrCa, with expression found in stromal tissue, and progression delayed in knockout mice [205,214].

The clear exception in this family was ADAM-19, which was found to be more highly expressed in normal tissue compared to PrCa and negatively correlated to the grade and relapse, with the enforced expression leading to decreased proliferation, metastatic ability and survival [208].

### 2.5. A Disintegrin and Metalloproteinase with Thrombospondin Motifs (ADAMTSs)

Eight studies were identified that related to the ADAMTS subgroup in PrCa progression (Table 5). The majority focused on ADAMTS-1, providing evidence of a tumor-suppressing function. ADAMTS-1 expression was typically decreased in PrCa samples, patients with metastatic disease, and a PrCa cell line variant with higher metastatic potential but elevated in slower-growing PrCa tumors in mice [65,179,219,220]. This was supported by functional data from the cells in which ADAMTS-1 expression had been modulated, which suggested a role in growth, although this appeared to depend on the cell line used [219]. ADAMTS-15 was also shown to be able to suppress tumor growth and migration, although it augmented survival [221]. Other members of the ADAMTS subgroup have also been shown to be expressed in PrCa cell lines, but their role in PrCa progression remains elusive [222].

## 3. Discussion

### 3.1. Overview

This study used a systematic-like review strategy to identify publications examining the role of metzincins in PrCa progression. While limited to articles in PUBMED and MEDLINE and those written in English, this approach was likely to yield the vast majority of relevant research publications. It is evident from a close examination of the 205 articles identified that the contributions made by members of the metzincin superfamily to PrCa disease progression are complex. For many individual members and, indeed, the entire Astracin family, there is currently no evidence of involvement. However, a significant number of metzincins are positively associated with PrCa, supported by functional evidence in a number of cases, while others were negatively associated with this disease. The positive associations were particularly strong with specific members of the MMP, MT-MMP and ADAM subgroups, while those within the ADAMTS subgroup or the important TIMP family of regulators were more likely to show negative associations.

### 3.2. Positive Associations

**Figure 2:**
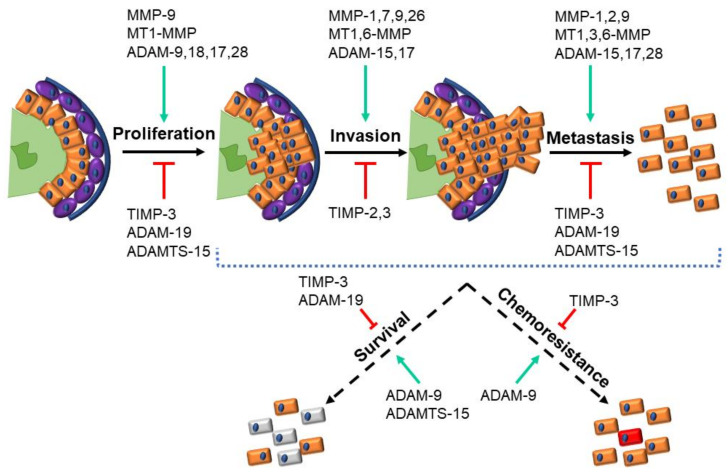
Metzincins and their regulators in prostate cancer. Schematic representation of prostate cancer progression highlighting the key cellular functions that are modulated by the indicated metzincin superfamily members, along with the regulatory Tissue Inhibitor of Metalloproteinase (TIMP) proteins (green: Lumen, orange: Luminal cells, purple: Basal cells, and blue: Basement membrane).

The clearest evidence for positive contributions to PrCa and its progression was for MMP-2, MMP-7, MMP-9, MT1-MMP, ADAM-15, ADAM-17 and ADAM-28, with supporting evidence for MMP-1, MT3-MMP, MT6-MMP and ADAM-9 (Table 1, Table 2 and Table 4). This is underpinned by studies that have identified associations between the expression and PrCa, which, in the case of MMP-7 expression [60,63,73,88,99,104,106,110,135,147] and ADAM-15 expression [190,199,200], correlated with the pathological stage and poorer outcomes for patients. This was supported by functional analyses that consistently identified enhancements in the proliferation, invasion/spread and metastasis/migration facilitated by them [56,106,110,200] (Figure 2). This identified these specific metzincins as likely tumor-promoting factors and so represented the obvious candidates as disease biomarkers or as potential targets for therapeutic agents.

### 3.3. Negative Associations

The strongest evidence for negative contribution to PrCa is for TIMP-2 and TIMP-3, as well as ADAM-19, ADAMTS-1 and ADAMTS-15 (Table 3, Table 4 and Table 5). Such a role for the ADAMTS proteins is somewhat counterintuitive, since these enzymes cleave ECM components like other metzincins [224], including those involved in PrCa disease progression [220,221]. However, the functional evidence points to these enzymes inhibiting key phenotypes, including proliferation and metastasis/migration, although not survival (Figure 2), presumably due to the different specificities for ECM components compared to other metzincins [220,221]. ADAM-19 was also implicated in the proliferation, metastasis/migration and survival (Figure 2), although this could relate to the known nonenzymatic functions for these enzymes. A negative role for the TIMP family was less surprising, given their primary role in the inhibition of MMP enzymes [225], with this impact extending across the entire gamut of relevant cell functions (Figure 2). These molecules can also be considered biomarker candidates to aid in prognosis. Therapeutic approaches targeting these proteins would likely be more limited, however, since they would need to augment, rather than inhibit, their function.

### 3.4. Mixed Associations

For other metzincins, the evidence for their involvement in PrCa was even more variable and contradictory, such as for MMP-11. Indeed, even those metzincins or regulators with consistent positive or negative correlations with PrCa were often reported in some studies to have no correlation or, indeed, the opposite correlation. This suggests a complex interplay between metzincins and PrCa.

### 3.5. Understanding the Complexity

In interpreting the variable and, at times, conflicting data, there are a number of factors that need to be considered. Firstly, different studies have utilized alternate approaches, such as analyzing the expression at the gene versus protein levels, that do not always correlate [24,68,82,83,91,101,140,189] or examining the enzymatic activity, which is not always reflective of metzincin expression [119,226], or, instead, considering the cellular localization [140]. Moreover, different samples have been analyzed in the literature, including plasma/serum, urine and tumor biopsies from PrCa patients, with several studies highlighting the differences between tissues [119], while the exact PrCa stage is also critical [117]. Other studies have employed PrCa cell lines and xenotransplanted tumors in mice, the relevance of which to human disease is assumed but not guaranteed. Secondly, it is clear that the factors controlling the expression of these enzymes and their regulators are complex. Thus, many metzincins have been demonstrated to be regulated by androgens [107,213,217,223,227,228], which can clearly be a complicating factor given the environment in which these cancers develop. In addition, expression is also impacted by oncogenes [85,147], inflammation and inflammatory cytokines [99,229], as well as angiogenic factors such as vascular endothelial factor [216], which are intrinsic features of any cancer. The cellular environment can further influence both expression [127] and activation [143]. Therefore, discerning the direction of causality between the expression and PrCa is not always straightforward.

In most cases, the effects of the metzincin superfamily member (or inhibitor) have been presumed to relate to the primary role for metzincins in regulating components of the ECM, which is known to be a particularly key element of metastasis [9,13]. However, which substrates are important? The cleavage of laminin [159,176], perlecan [56] and beta-4 integrin [230] have all been shown to correlate with the effects of protumorigenic metzincins, particularly on metastasis, whereas versican has been identified as a target of the antitumorigenic ADAMTS-15 [221]. Clearly, more research is required to understand this important aspect of metzincin pathobiology. Moreover, other roles should also be considered, especially given reports suggesting that nuclear localization may be important in some situations [204,213,216], with both ADAMs and ADAMTSs known to have nonenzymatic roles.

There also remains a lack of depth in our understanding of how metzincins are regulated at the protein level, including by other metzincins. TIMPs are clearly important for the negative regulation of MMPs [114]. TIMPs are typically downregulated as cancer progresses and can act as independent correlates of PrCa progression [25,32,95,112,114,116,177,178,179], especially when combined with MMP expression [83,105,116]. TIMP-2 and TIMP-3 have also been shown to inhibit ADAMTS-1 [185]. Are there equivalents for ADAM and other ADAMTS enzymes? In addition, MT1-MMP has been shown to exert its role at least in part through the direct activation of MMP-2 [140,159]. Is this crosstalk common across metzincins? More research is needed to gain further insight in this area.

## 4. Materials and Methods

This study represents a systematic-like review of the role of the metzincin superfamily of proteases in PrCa progression. The search terms were identified through a PCC (population, context and concept) format by the research team with keywords, Boolean operators, truncations and Medical Subject Headings (MeSH) used to develop a database search strategy in collaboration with a specialist health librarian. In reporting the review, the Preferred Reporting Items for Systematic Reviews and Meta-analysis (PRISMA) was utilized.

### 4.1. Search Strategy

A preliminary search was undertaken using MEDLINE and then a full search run through both the PUBMED and MEDLINE databases.

### 4.2. Inclusion and Exclusion Criteria

**Figure 3:**
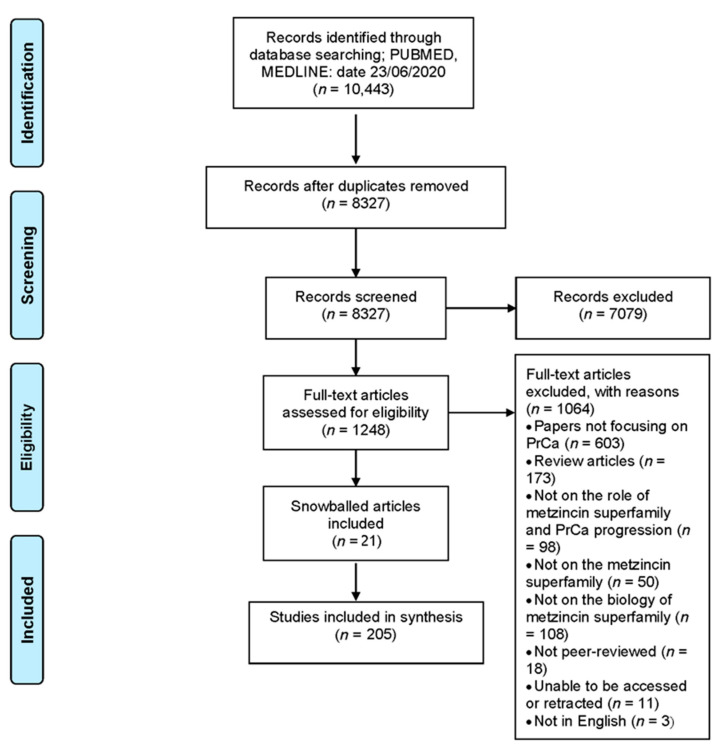
Preferred Reporting Items for Systematic Reviews and Meta-analysis (PRISMA) overview of the systematic-like review. Flow chart of the systematic-like review process undertaken, including the details of identification, screening and assessment for eligibility.

All studies were considered based on the inclusion and exclusion criteria shown in Table 6. Search terms for inclusion were “metzinzin”, “metalloproteases”, “metalloproteinase”, “MMP”, “TIMP”, “ADAM”, “ADAMTS”, “BMP1” or “meprin” and “neoplasm”, “neoplasia”, “cancer”, “tumor” or “cysts”. Reasons for exclusion after the full-text review are detailed in Figure 3. No restrictions were put on the date that articles were published.

### 4.3. Study Selection and Data Extraction

Searches of the published literature were conducted by M.J.B. in collaboration with a specialist health librarian. Titles and abstracts were retrieved from the search and screened. Full-text article review and data extraction was then conducted, with the reasons for exclusion documented. The reference lists of the included articles were also reviewed to identify further potential articles for inclusion in the review.

### 4.4. Data Analysis

Database searching identified 10,443 publications. After duplicate removal, the titles and abstracts from 8327 were reviewed against the inclusion criteria. Full-text versions of 1248 articles were then further reviewed, identifying 205 articles for inclusion (Figure 2). The reasons for exclusion were a lack of focus on PrCa (*n* = 603) or the metzincin superfamily (*n* = 50) or the role of the metzincin superfamily family in PrCa progression (*n* = 98) or on the biology of the metzincin superfamily (*n* = 108) or not peer reviewed (*n* = 18) or being review articles (*n* = 173) or articles unable to be accessed or retracted (*n* = 11) or not in English (*n* = 3). The 205 included articles covered members of the Matrixin family subgroups MMP and MT-MMPs, the TIMPs and the Adamalysin family subgroups ADAMs and ADAMTSs, but there were none regarding the Astracin family subgroups BMP/TLL or Meprin.

## Figures and Tables

**Table 1:** Studies on soluble Matrix Metalloproteinases (MMPs) in prostate cancer (PrCa).

Authors	Year	MMP	PrCa Platform	Role
Aalinkeel et al. [20]	2004	MMP-9 *	Yes—Prostate cell lines	MMP-9 expression increased in metastatic PrCa lines. Enforced expression of MMP-9 increased invasiveness, whereas ablation of expression decreased invasiveness.
Aalinkeel et al. [21]	2011	MMP-9	Yes—Prostate cell lines	Enforced expression of MMP-9 increased invasiveness, whereas ablation decreased invasiveness, with no change in migration.
Adabi et al. [22]	2015	MMP-2	Yes—Prostate samples	MMP-2 polymorphism not associated with PrCa risk or degree of metastasis.
Albayrak et al. [23]	2007	MMP-1	Yes—Prostate samples	MMP-1 polymorphism not associated with PrCa risk.
Assikis et al. [24]	2004	MMP-9	Yes—Prostate samples	MMP-9 expression low in PrCa.
Babichenko et al. [25]	2014	MMP-9*	Yes—Prostate samples	MMP-9 expression negatively correlated with Gleason score and proliferation index.
Baspinar et al. [26]	2017	MMP-9	Yes—Prostate samples	MMP-9 expression increased in samples with high metastatic potential scores and staging.
Bekes et al. [27]	2011	MMP-9	Yes—Prostate cell lines	Increased MMP-9-positive neutrophils in highly disseminated PrCa correlated with angiogenic potential.
Białkowska et al. [28]	2018	MMP-1, 2, 7 and 13	Yes—Prostate samples	Polymorphism in MMP-7 (but not MMP-1, 2 and 13) correlated with increased PrCa risk.
Bok et al. [29]	2003	MMP-2, 3, 7& 9	Yes-Mice	Active forms of MMP-2 and 9 present in late stage PrCa in mouse model, but MMP-3 not expressed and MMP-7 only focal expression.
Bonaldi et al. [30]	2015	MMP-13	Yes—Prostate samples	MMP-13 expression not changed in PrCa compared to healthy controls.
Boxler et al. [31]	2010	MMP-2, 3, 7, 9, 13 and 19	Yes—Prostate samples	Expression of MMP-9 (but not MMP-2, 3, 7, 13 or 19) negatively correlated with overall, recurrence-free and disease-specific survival.
Brehmer et al. [32]	2003	MMP-2 and 9 *	Yes—Prostate samples	MMP-2 expressed significantly in more advanced PrCa tumors, and MMP-9 significantly less.
Bruni-Cardoso et al. [33]	2010	MMP-9	Yes—Mice and rats	MMP-9 expression in osteoclasts contributed to PrCa tumor growth in the bone through increased angiogenesis.
Cardillo et al. [34]	2006	MMP-1, 7 and 9 *	Yes—Prostate samples	Expression of MMPs significantly increased in the epithelium than the stroma, and of MMP-7 and 9 (but not MMP-1) with Gleason score.
Carozzi et al. [35]	2016	MMP-2 and 9	Yes—Prostate samples	Expression of MMP-2 and 9 negatively correlated with survival.
Castellana et al. [36]	2009	MMP-2, 3, 7, 9 and 13 *	Yes—Prostate cell lines	Tumor-derived microvesicles induced MMP-9 expression that correlated with increased migration and resistance to apoptosis.
Coulson-Thomas et al. [37]	2010	MMP-1 and 9 *	Yes—Prostate cell lines	MMP-1 and 9 differentially expressed following co-culture with metastatic PrCa.
Daja et al. [38]	2003	MMP-1 and 13 *	Yes—Prostate cell lines	MMP-1 and 13 expression higher in more aggressive sublines
De Cicco et al. [39]	2008	MMP-2 and 9 *	Yes—Prostate samples	Low serum MMP-2 (but not MMP-9) associated with increased risk of disease progression.
Di Carlo et al. [40]	2010	MMP-2 and 9	Yes—Prostate samples/urine	Active MMP-9 in urine (but not MMP-2) decreased in PrCa versus benign prostatic hyperplasia.
Dong et al. [41]	2001	MMP-9	Yes—Prostate cell lines and mice	Pro-MMP-9 expression levels enhanced during PrCa co-culture, including in bone implants in mice.
dos Reis et al. [42]	2009	MMP-1, 2, 7 and 9	Yes—Prostate samples	Polymorphisms in MMP-1, 2 and 9 (but not MMP-7) lower in PrCa versus controls.
dos Reis et al. [43]	2010	MMP-2	Yes—Prostate samples	Polymorphism in MMP-2 more frequent in PrCa, including in higher Gleason scores, compared to those in MMP-9 that were associated with lower scores.
dos Reis et al. [44]	2008	MMP-1, 2, 7 and 9	Yes—Prostate samples	Polymorphisms in MMP-1, 2 and 9 (but not MMP-7) lower in PrCa versus controls.
Eiro et al. [45]	2017	MMP-2, 9 and 11	Yes—Prostate samples	MMP-2 expression lower and MMP-11 higher in cancer-associated fibroblasts in PrCa.
El-Chaer et al. [46]	2020	MMP-1	Yes—Prostate samples/Serum	Genotype adjusted MMP-1 expression higher in PrCa compared to benign prostatic hyperplasia.
Eryilmaz et al. [47]	2019	MMP-2 and 9	Yes—Prostate samples	MMP-2 expression associated with increased PrCa risk.
Escaff et al. [48]	2010	MMP-1, 2, 7, 9, 11 and 13 *	Yes—Prostate samples	Increased expression of MMP-11 and 13 associated with significant probability of biochemical recurrence.
Escaff et al. [49]	2011	MMP-1, 2, 7, 9, 11 and 13 *	Yes—Prostate samples	Expression of MMP-2 in fibroblasts and MMP-9 in mononuclear inflammatory cells associated with PrCa.
Escaff et al. [50]	2011	MMP-1, 2, 7, 9, 11 and 13 *	Yes—Prostate samples	Expression of MMP-9 and 13 in fibroblasts. MMP-13 in tumor cells associated with biological recurrence.
Favaro et al. [51]	2012	MMP-2	Yes—Prostate samples	MMP-2 expression increased in periacinar retraction during PrCa.
Fernandez-Gomez et al. [52]	2011	MMP-1, 2, 7, 9, 11 and 13 *	Yes—prostate samples	Expression of MMP-2 negatively associated with high tumor grade, MMP-7 expression negatively associated with Prostate-Specific Antigen (PSA), whereas MMP-13 expression positively associated with PSA.
Festuccia et al. [53]	1996	MMP-2 and 9	Yes—Prostate samples and prostate cell lines	MMP-2 and 9 highly expressed in PrCa. High MMP-9 expression and activity relative to MMP-2 associated with high Gleason grade.
Gohji et al. [54]	1998	MMP-2	Yes—Prostate samples/Serum	Serum MMP-2 higher in patients with PrCa and higher in those with metastasis.
Gravina et al. [55]	2013	MMP-2 and 9 *	Yes—Prostate cell lines	MMP-2 consistently secreted by PrCa, whereas MMP-9 secretion sporadic.
Grindel et al. [56]	2014	MMP-7	Yes—Prostate cell lines	MMP-7 expression associated with increased invasiveness.
Gupta et al. [57]	2013	MMP-9	Yes—Prostate cell lines	MMP-9 knockdown resulted in increased adhesion and cell spreading.
Hamdy et al. [58]	1994	MMP-9	Yes—Prostate samples	MMP-9 activity increased in malignant PrCa tissue compared to benign.
Hanqing et al. [59]	2003	MMP-2 and 9	Yes—Prostate samples	MMP-2 and 9 expression higher in PrCa tissue.
Hashimoto et al. [60]	1998	MMP-7 (matrilysin) *	Yes—Prostate samples	MMP-7 levels and MMP-7/TIMP-1 ratio higher in advanced PrCa, and correlated with pathological stage, lymph node metastasis, histological differentiation, as well as vascular and lymphatic invasion.
Hetzl et al. [61]	2012	MMP-2 and 9	Yes—Prostate samples	MMP-2 and 9 expression increased in PrCa versus controls.
Incorvaia et al. [62]	2007	MMP-2 and 9	Yes—Prostate samples	Circulating MMP-9 (but not MMP-2) showed significant correlation with PSA.
Jaboin et al. [63]	2011	MMP-7 (matrilysin)	Yes—Prostate samples	MMP-7 polymorphism associated with PrCa recurrence.
Jędroszka et al. [64]	2017	MMP-2, 3 and 9	Yes—Prostate samples	Expression of MMP-2, 3 and 9 increased in Gleason grade 8 and 9 tissues.
Jennbacken et al. [65]	2006	MMP-2 and 9 *	Yes—Prostate cell lines	MMP-9 expression increased in PrCa, but MMP-2 expression not detected.
Jung et al. [66]	1998	MMP *	Yes—Prostate samples	MMP levels decreased but MMP/TIMP ratio increased in PrCa.
Jung et al. [67]	2003	MMP-2 and 9	Yes—Rats	Expression of MMP-9 (but not MMP-2) increased in advanced PrCa.
Jung et al. [68]	1997	MMP-1 and 3 *	Yes—Prostate samples	MMP-3 (but not MMP-1) highly expressed in PrCa patients with metastasis.
Jurasz et al. [69]	2003	MMP-2	Yes—Prostate samples/Serum	Platelet MMP-2 levels increased in metastatic versus localized PrCa.
Kalantari et al. [70]	2019	MMP-13	Yes—Prostate samples	MMP-13 highly expressed in PrCa tissue and associated with Gleason score.
Kaminski et al. [71]	2006	MMP-1	Yes—Prostate cell lines	PrCa conditioned medium increased MMP-1 expression in fibroblasts.
Kanoh et al. [72]	2002	MMP-2	Yes—Prostate samples/Serum	Serum MMP-2 increased in PrCa and bone metastasis, but not correlated with PSA.
Knox et al. [73]	1996	MMP-7 (matrilysin)	Yes—Prostate samples	MMP-7 expressed in PrCa.
Koshida et al. [74]	2004	MMP-1 and 2	Yes—Prostate cell lines	MMP-1 and 2 expressed in PrCa, but only MMP-2 expression increased following implantation.
Kuniyasu et al. [75]	2000	MMP-2 and 9	Yes—Prostate samples	Expression of MMP-2 and 9 in high grade tumors and associated with Gleason score.
Kuniyasu et al. [76]	2003	MMP-2 and 9	Yes—Prostate samples	Increased MMP/E-cadherin ratio correlated with increased stage.
Larsson et al. [77]	2020	MMP-9	Yes—Prostate samples, Prostate cell lines and mice	High MMP-9 expression associated with poor prognosis.
Latil et al. [78]	2003	MMP-9 *	Yes—Patient samples	MMP-9 expressed in PrCa tissue.
Lein et al. [79]	1999	MMP-2 and 3 *	Yes—Prostate samples/Serum	Plasma MMP-3 (but not MMP-2) increased in PrCa.
Leshner et al. [80]	2016	MMP-2 and 9 *	Yes—Prostate samples	MMP-9 gene repositioned in PrCa and MMP-2 in both PrCa and hyperplasia.
Liao et al. [81]	2018	MMP-1	Yes—Prostate samples/Serum	MMP-1 promotor polymorphisms not a risk factor for PrCa.
Lichtinghagen et al. [82]	2002	MMP-2 and 9 *	Yes—Prostate samples	Expression of MMP-2 gene decreased and MMP-9 unchanged, but MMP-9 protein higher in cancerous tissue, with no change in MMP-2 protein.
Lichtinghagen et al. [83]	2003	MMP-1, 2, 7, 9 and 11 *	Yes—Prostate samples	Expression of MMP-2 and 11 decreased, and MMP-9 increased in PrCa, but no correlations with grade, stage or PSA.
Littlepage et al. [84]	2010	MMP-2, 7, 9 and 13 *	Yes—Mice	Expression of MMP-2, 7 and 9 increased with PrCa progression. MMP-2 knockout mice showed reduced tumor burden, prolonged survival, decreased lung metastasis, and decreased blood vessel density. Knockout of MMP-7 or MMP-9 did not impact tumor growth or survival but affected blood vessel formation.
Liu et al. [85]	2017	MMP-9	Yes—Prostate samples and prostate cell lines	MMP-9 expression increased in metastatic cancer.
Lokeshwar et al. [86]	1993	MMP-2 and 9 *	Yes—Prostate samples/Serum	MMP-2 and 9 secretion, including of the active form of MMP-2, increased in neoplastic tissue.
London et al. [87]	2003	MMP-9	Yes—Prostate cell lines	Ablation of MMP-9 caused decreased tumor invasion, migration, and growth.
Lynch et al. [88]	2005	MMP-7 (matrilysin)	Yes—Rat	MMP-7 expression increased at tumor/bone interface. MMP-7 knockout mice showed reduced tumor-induced osteolysis.
Marin-Aguilera et al. [89]	2015	MMP-9	Yes—Prostate samples	MMP-9 upregulated in PrCa and correlated with poorer overall survival.
Maruta et al. [90]	2010	MMP-10	Yes—Prostate samples	MMP-10 expression correlated with stage, cell renewal and vascular invasion.
Medina-González et al. [91]	2020	MMP-2, 9, 11 and 13 *	Yes—Prostate samples	Expression of MMP-2 and 9 increased (but MMP-11 and 13 unchanged) in PrCa.
Miyake et al. [92]	2010	MMP-2 and 9	Yes—Prostate samples	MMP-2 and 9 expression correlated with stage, recurrence, proliferation, and invasion.
Montironi et al. [93]	1995	MMP-2 (type IV collagenase) *	Yes—Prostate samples	MMP-2 protein expression identified in cells in contact with the stroma
Montironi et al. [94]	1996	MMP-2 (type IV collagenase)	Yes—Prostate samples	MMP-2 expression correlated with progression.
Morgia et al. [95]	2005	MMP-2, 3 and 13 *	Yes—Prostate samples	Plasma levels of MMP-2 and 9 (but not MMP-13) increased in metastatic PrCa.
Moses et al. [96]	1998	MMP-2 and 9	Yes—Prostate samples/Urine	Active MMP-2 and 9 in urine were independent predictor of organ-confined PrCa.
Muñoz et al. [97]	2017	MMP-2 and 9	Yes—Prostate samples/Urine	No difference in urine levels of MMP-2 or MMP-9 species in PrCa.
Nabha et al. [98]	2006	MMP-9	Yes—Mice	MMP-9 knockout resulted in no difference in tumor incidence, growth or microvascularity.
Nagle et al. [99]	1994	MMP-7 (matrilysin)	Yes—Prostate samples	MMP-7 expression in PrCa located in dilated ducts when inflamed and atrophic glands.
Nalla et al. [100]	2010	MMP-9	Yes—Prostate cell lines	Ablation of MMP-9 reduced migration and invasion and induced apoptosis.
Neuhaus et al. [101]	2017	MMP-3, 7, 13 and 20 *	Yes—Prostate samples	Decreased MMP-3/TIMP ratio in PrCa, but other MMPs not altered.
Oguić et al. [102]	2014	MMP-2 and 9	Yes—Prostate samples	Higher MMP-2 and 9 expression in positive surgical margins. MMP-9 expression associated with biochemical recurrence.
Ok Atılgan et al. [103]	2020	MMP-9	Yes—Patient samples	MMP-9 expression positively associated with WHO grade, tumor stage, extracapsular extension, positive surgical margin lymphovascular, perineural invasion and decreased disease-free survival.
Ouyang et al. [104]	2001	MMP-7 (matrilysin)	Yes—Rats	MMP-7 expressed in premalignant and malignant tissue.
Ozden et al. [105]	2013	MMP-1 and 9 *	Yes—Prostate samples	MMP-1 expression in tumors correlated with higher grades and Gleason scores. MMP-9 expression in normal glands correlated with low PSA and Gleason scores.
Pajouh et al. [106]	1991	MMP-7 (matrilysin)	Yes—Prostate cell lines	MMP-7 expressed in invasive metastatic primary human PrCa.
Pang et al. [107]	2004	MMP-13	Yes—Prostate samples and prostate cell lines	MMP-13 expressed in PrCa.
Pettaway et al. [108]	2008	MMP-2 and 9	Yes—Prostate samples	MMP-2 and MMP-9/E-cadherin ratio increased at tumor edge and correlated with disease, biochemical recurrence, and pathological stage.
Pouyanfar et al. [109]	2016	MMP-9	Yes—Prostate samples	MMP-9 expression higher in PrCa patients related to Gleason score and age, but not PSA, metastasis, or survival.
Powell et al. [110]	1993	MMP-7 (matrilysin)	Yes—Prostate cell lines and mice	Enforced MMP-7 expression led to increased invasion.
Prior et al. [111]	2010	MMP-2	Yes—Prostate samples	Increased MMP-2 levels in urine/blood associated with PrCa progression.
Reis et al. [112]	2012	MMP-2 *	Yes—Prostate samples	MMP-2 expression reduced in PrCa samples but increased in higher grades.
Reis et al. [113]	2015	MMP-2&9 *	Yes—Prostate samples	MMP-2 and 9 expressed in most PrCa but no prognostic value.
Reis et al. [114]	2011	MMP-9 *	Yes—Prostate samples	Higher MMP-9 expression associated with increased PSA and recurrence, but not Gleason score.
Riddick et al. [115]	2005	MMP-2, 10, 23 and 25 *	Yes—Prostate samples	Increased expression of MMP-10 and 25, but MMP-2 and 23 decreased in PrCa.
Ross et al. [116]	2003	MMP-2 *	Yes—Prostate samples	MMP-2 expressed in more advanced PrCa and correlated with prognostic variables.
Sakai et al. [117]	2005	MMP-2 and 9	Yes—Prostate samples	Increased expression of MMP-2 and 9 in peripheral zone cancers compared to transitional zone.
San Francisco et al. [118]	2004	MMP-9	Yes—Prostate cell lines	MMP-9 expression not changed in PrCa.
Sauer et al. [119]	2004	MMP-2 and 9	Yes—Prostate samples/Serum	MMP-9 serum levels increased in PrCa patients and correlated with grade, but tissue MMP-9 activity not related to stage or grade. MMP-2 activity correlated with disease progression.
Schäfer et al. [120]	2012	MMP-9	Yes—Prostate cell lines and mice	Enforced MMP-9 expression enhanced tumor regression and impacted metastasis.
Schveigert et al. [121]	2013	MMP-9	Yes—Prostate samples	MMP-9 polymorphism and increased expression associated with PrCa, with polymorphism related to pathological stage and prognostic group, and expression with survival.
Sehgal et al. [122]	1998	MMP-9	Yes—Mice	Ablation of MMP-9 reduced metastatic potential.
Sehgal et al. [123]	2003	MMP-1, 2 and 9 *	Yes—Prostate cell lines	Expression of MMP-1 (but not MMP-2 or MMP-9) decreased in more metastatic PrCa.
Serretta et al. [124]	2018	MMP-3	Yes—Prostate samples	MMP-3 expression not associated with Gleason score 4 and 5.
Sfar et al. [125]	2007	MMP-9	Yes—Prostate samples	MMP-9 polymorphism associated with increased risk of advanced PrCa.
Sfar et al. [126]	2009	MMP-9	Yes—Prostate samples	MMP-9 polymorphism associated with increased risk of developing PrCa.
Shah et al. [127]	2016	MMP-9	Yes—Prostate cell lines	Increased MMP-9 expression in PrCa cells co-cultured with dermal lymphatic microvascular endothelial cells.
Shajarehpoor Salavati et al. [128]	2017	MMP-2	Yes—Prostate sample	MMP-2 polymorphism not associated with PrCa risk.
Shi et al. [129]	2017	MMP-9	Yes—Prostate samples/Urine	MMP-9 detected in urine of PrCa patients.
Silva et al. [130]	2015	MMP-2	Yes—Prostate samples	Increased MMP-2 expression in reactive stroma.
Srivastava et al. [131]	2012	MMP-2 *	Yes—Prostate samples	MMP-2 polymorphism associated with PrCa risk but not staging.
Stearns et al. [132]	1996	MMP-2	Yes—Prostate samples and prostate cell lines	MMP-2 expression increased in higher PrCa grades.
Stearns et al. [133]	1996	MMP-2	Yes—Prostate samples	MMP-2a expressed in glandular epithelial cells and increased in PrCa samples with high Gleason score.
Still et al. [134]	2000	MMP-2 *	Yes—Prostate samples	MMP-2 localized to malignant cells, with increased MMP-2/TIMP-2 ratio associated with high grade and stage.
Szarvas et al. [135]	2018	MMP-7 (matrilysin)	Yes—Prostate samples	Serum MMP-7 level significantly higher in docetaxel-resistant PrCa and associated with poor survival.
Trudel et al. [136]	2008	MMP-2 *	Yes—Prostate samples	MMP-2 expression in basal epithelial cells and stromal cells associated with shorter disease-free survival.
Trudel et al. [137]	2003	MMP-2	Yes—Prostate samples	MMP-2 expression in malignant cells associated with disease-free survival.
Trudel et al. [138]	2010	MMP-9	Yes—Prostate samples	MMP-9 expression correlated with Gleason score but not disease-free survival.
Tsuchiya et al. [139]	2009	MMP-1	Yes—Prostate samples	MMP-1 promotor polymorphisms (and increased expression) associated with pathological stage but not PrCa susceptibility or progression.
Upadhyay et al. [140]	1999	MMP-2 *	Yes—Prostate samples	MMP-2 localization altered in PrCa.
Vallbo et al. [141]	2005	MMP-2 and 9	Yes-Rat	MMP-2 (but not MMP-9) expressed in malignant cells.
Wang et al. [142]	2014	MMP-1	Yes—Prostate samples	MMP-1 expression significantly increased in PrCa and associated with higher Gleason score, metastasis and pathological stage, as well as reduced overall and recurrence-free survival.
Wiesner C [143]	2007	MMP-9	Yes—Prostate cell lines and mice	Increased MMP-9 activity when PrCa cells interact with bone.
Wilson et al. [144]	2002	MMP-2 and 9	Yes—Prostate samples and prostate cell lines	Epithelial cells secreted little MMP-2 or MMP-9, whereas pro-MMP-2 (but not MMP-9) secreted by stromal cells.
Wilson et al. [145]	1993	MMP	Yes—Prostate cell lines and mice	MMP isoforms differentially altered in PrCa.
Wood et al. [146]	1997	MMP-2 and 9 *	Yes—Prostate samples	Increased expression of MMP-2&9 in high grade samples.
Xie et al. [147]	2016	MMP-7 (matrilysin)	Yes-Mice	MMP-7 expression elevated in PrCa.
Xu et al. [148]	2010	MMP-9	Yes—Prostate cell lines and mice	Ablation of MMP-9 led to decreased cell invasion.
Yaykaşli et al. [149]	2014	MMP-2 *	Yes—Prostate samples	MMP-2 polymorphisms increased in PrCa patients.
Zellweger et al. [150]	2005	MMP-2	Yes—Prostate samples	MMP-2 expressed in PrCa, but not altered across different types.
Zhang et al. [151]	2002	MMP-2 and 7 *	Yes—Prostate samples and prostate cell lines	MMP-2 expressed in stromal cells and MMP-7 expressed in epithelial cells. Differential expression between cell lines.
Zhang et al. [152]	2004	MMP-2 and 9	Yes—Prostate samples and Prostate cell lines	Expression of MMP-9 (but not MMP-2) increased in malignant samples.
Zhang et al. [153]	2008	MMP-2 and 9	Yes—Prostate samples and prostate cell lines	Expression of MMP-2 and 9 increased in PrCa.
Zhang et al. [154]	2018	MMP-1	Yes—Prostate cell lines	Expression of MMP-1 increased in more metastatic lines. Ablation of MMP-1 decreased invasion and migration.
Zhao et al. [155]	2003	MMP-9 and 26	Yes—Prostate samples and prostate cell lines	Expression of MMP-26 (but not MMP-9) increased in PrCa. Blocking of either reduced invasion.
Zhong et al. [156]	2008	MMP-1, 2 and 9	Yes—Prostate samples	MMP-1, 2 and 9 expression significantly higher in PrCa. MMP-2 expression correlated with TMN grade and Gleason score.
Zhu et al. [157]	1999	MMP-2 and 9	Yes—Prostate cell lines	MMP-2 expression enhanced when PrCa co-cultured.

* Included in at least one other table.

**Table 2:** Studies on Membrane-Tethered Matrix Metalloproteinase (MT-MMPs) in PrCa.

Authors	Year	MT-MMP	PrCa Platform	Role
Aalinkeel et al. [20]	2004	MT1- and MT4-MMP *	Yes—Prostate cell lines	MT4-MMP expression higher in metastatic PrCa cells lines.
Bair et al. [159]	2005	MT1-MMP	Yes—Prostate samples and prostate cell lines	Ablation of MT1-MMP decreased migration and invasion.
Bonfil et al. [163]	2007	MT1-MMP	Yes—Prostate samples and prostate cell lines	MT1-MMP expressed in PrCa bone metastasis. Enforced expression of MT1-MMP enhanced tumor growth and osteolysis.
Cao et al. [158]	2008	MT1-MMP	Yes—Prostate cell lines	MT1-MMP expression increased in PrCa. Enforced expression of MT1-MMP induced epithelial to mesenchymal transition associated with metastatic ability.
Cardillo et al. [34]	2006	MT1-MMP *	Yes—Prostate samples	MT1-MMP expression increased in epithelial and stromal tissues in PrCa.
Castellana et al. [36]	2009	MT1-MMP *	Yes—Prostate cell lines	MT1-MMP protein levels high in PrCa microvesicles.
Cheng et al. [165]	2017	MT6-MMP	Yes—Prostate samples/Serum	MT6-MMP expression upregulated in serum and tissue in PrCa.
Chu et al. [166]	2006	MT3-MMP	Yes—Prostate cell lines and mice	MT3-MMP expressed in PrCa tumors, especially in lymph node metastases.
Coulson-Thomas et al. [37]	2010	MT1-MMP *	Yes—Prostate cell lines	MT1-MMP expressed in the stromal cells during co-culture with metastatic PrCa cells, extending into the ECM
Daja et al. [38]	2003	MT1-, MT2- and MT3-MMP *	Yes—Prostate cell lines	MT1&3-MMP (but not MT2-MMP) expressed highly, particularly processed versions, in aggressive PrCa cell lines.
Jennbacken et al. [65]	2006	MT1-MMP *	Yes—Prostate cell lines	MT1-MMP expression increased in more invasive PrCa subline.
Jiang et al. [167]	2017	MT3-MMP	Yes—Prostate samples and prostate cell lines	High levels of MT3-MMP associated with advanced tumor stage and metastasis. Ablation of MT3-MMP decreased migration.
Jung et al. [168]	2003	MT1- and MT5-MMP	Yes—Prostate samples and prostate cell lines	MT1 and 5-MMP expressed in most PrCa cell lines and prostate tissue, with variable expression in metastatic lines and malignant tumors, with no correlation to tumor classification.
Khamis et al. [169]	2016	MT6-MMP	Yes—Prostate samples and prostate cell lines	MT6-MMP expression up-regulated in high grade prostate intraepithelial neoplasia but decreased with PrCa progression, with MT6-MMP expressing cells prone to apoptosis.
Latil et al. [78]	2003	MT1-MMP *	Yes—Patient samples	MT1-MMP expression decreased in PrCa tissue.
Lee et al. [170]	2006	MT6-MMP	Yes—Prostate samples	MT6-MMP expression increased in high-grade prostatic intraepithelial neoplasia but reduced in invasive cancer.
Lichtinghagen et al. [83]	2003	MT1-MMP *	Yes—Prostate samples	MT1-MMP expression observed in PrCa, but no correlation with grade, stage, or serum PSA.
Lin et al. [171]	2013	MT3-MMP	Yes—Prostate samples	MT3-MMP single nucleotide polymorphisms associated with PrCa aggressiveness.
Lin et al. [172]	2016	MT3-MMP	Yes—Prostate samples	MT3-MMP expression associated with PrCa aggressiveness.
Littlepage et al. [84]	2010	MT1-MMP *	Yes—Mice	Broad MMP inhibitor reduced tumor burden.
Liu et al. [173]	2010	MT1-MMP	Yes—Prostate cell lines	MT1-MMP ablation reduced susceptibility to immune-mediated killing.
Nagakawa et al. [174]	2000	MT1-MMP	Yes—Prostate cell lines	MT1-MMP expression increased in more metastatic PrCa lines.
Neuhaus et al. [101]	2017	MT1-MMP *	Yes—Prostate samples	MT1-MMP expression decreased in PrCa but increased in benign prostatic hyperplasia.
Nguyen et al. [161]	2011	MT1-MMP	Yes—Prostate cell lines	Enforced MT1-MMP expression increased invasion.
Reis et al. [112]	2012	MT1-MMP *	Yes—Prostate samples	MT1-MMP under-expressed in PrCa.
Riddick et al. [115]	2005	MT2-, MT5- and MT6-MMP *	Yes—Prostate samples	MT2- and MT6-MMP (but not MT5-MMP) expression correlated positively with Gleason score.
Sabbota et al. [160]	2010	MT1-MMP	Yes—Prostate cell lines	Enforced MT1-MMP expression enhanced tumor migration.
Sroka et al. [175]	2008	MT1-MMP	Yes—Prostate samples and prostate cell lines	MT1-MMP expression in apical regions in PIN and PrCa.
Udayakumar et al. [176]	2003	MT1-MMP	Yes—cells and patients	Ablation of MT1-MMP enhanced cell migration.
Upadhyay et al. [140]	1999	MT1-MMP *	Yes—Prostate samples	MT1-MMP localized in benign glands changing to cytoplasmic staining and then heterogenous as PrCa progressed. Increased vasculature when MT1-MMP co-localized with MMP-2.
Wang et al. [164]	2009	MT1-MMP	Yes—Prostate cell lines and mice	MT1-MMP expression increased in tumor cells.Enforced expression increased tumor growth in mice.
Zarrabi et al. [162]	2011	MT1-MMP	Yes—Prostate cell lines	MT1-MMP inhibition decreased cell migration, but not growth.
Zhang et al. [151]	2002	MT1- and MT3-MMP *	Yes—Prostate samples and prostate cell lines	MT1 and 3-MMP expressed in stromal and epithelial cells in PrCa.
Zhao et al. [155]	2003	MT6-MMP *	Yes—Prostate samples and prostate cell lines	MT6-MMP highly expressed in PrCa samples. Inhibition of MT6-MMP decreased invasion.

* Included in at least one other table.

**Table 3:** Studies on the Tissue Inhibitor of Metalloproteinases (TIMPs) in PrCa.

Authors	Year	TIMP	PrCa Platform	Role
Aalinkeel et al. [20]	2004	TIMP-1, 3 and 4 *	Yes—Prostate cell lines	TIMP-1 and 4 (but not TIMP-3) expressed higher in more metastatic PrCa cells.
Adissu et al. [181]	2015	TIMP-3	Yes—Mice	TIMP-3 mouse knockout exhibited enhanced PrCa tumor growth and invasion.
Ashida et al. [178]	2004	TIMP-1	Yes—Prostate samples	TIMP-1 expression down-regulated in the transition to PrCa.
Babichenko et al. [25]	2014	TIMP-1 *	Yes—Prostate samples	TIMP-1 expression lower in PrCa adenocarcinoma compared to benign prostatic hyperplasia.
Baker et al. [182]	1994	TIMP-1 and 2 *	Yes—Prostate samples	TIMP-1 expression higher and TIMP-2 lower in PrCa patients.
Brehmer et al. [32]	2003	TIMP-1 and 2 *	Yes—Prostate samples	TIMP-1 expression decreased in PrCa compared to normal tissue, whereas TIMP-2 expression not significantly different.
Daja et al. [38]	2003	TIMP-1 *	Yes—Prostate cell lines	TIMP-1 expression higher in more aggressive PrCa cell lines.
De Cicco et al. [39]	2008	TIMP-1 and 2 *	Yes—Prostate samples/Serum	TIMP-1 and 2 serum levels do not correlate with PrCa progression.
Deng et al. [183]	2006	TIMP-3	Yes—Prostate cell lines	Enforced TIMP-3 expression caused apoptosis and increased sensitivity to chemotherapeutic agents.
Escaff et al. [48]	2010	TIMP-1, 2 and 3 *	Yes—Prostate samples	TIMP-1 expression significantly increased in PrCa and associated with Gleason score.
Escaff et al. [49]	2011	TIMP-1, 2 and 3 *	Yes—Prostate samples	Expression of TIMP-3 (but not TIMP-1 and 2) increased in mononuclear inflammatory cells in PrCa carcinoma.
Fernandez-Gomez et al. [52]	2011	TIMP-1, 2 and 3 *	Yes—Prostate samples	TIMP-2 expression in mononuclear inflammatory cells significantly associated with decreased tumor grade. TIMP-3 expression in stromal fibroblasts correlated with histological grade.
Gong et al. [184]	2015	TIMP-1	Yes—Prostate samples and prostate cell lines	TIMP-1 highly expressed in tumors from castration-resistant PrCa patients.
Gravina et al. [55]	2013	TIMP-1, 2 and 3 *	Yes—Prostate cell lines	TIMP-1, 2 and 3 expression reduced in PrCa compared to benign prostatic hyperplasia.
Gustavsson et al. [185]	2008	TIMP-2 and 3 *	Yes—Prostate cell lines and mice	Expression of TIMP-2 (but not TIMP-3) higher in PrCa xenografts.
Hashimoto et al. [60]	1998	TIMP-1 *	Yes—Prostate samples	MMP-7/TIMP-1 ratio higher in advanced PrCa and correlated with increased invasion and elevated PSA.
Hoque et al. [186]	2005	TIMP-3	Yes—Prostate samples/Urine	TIMP-3 gene promoter methylated in urine samples of PrCa patients.
Jerónimo et al. [187]	2004	TIMP-3	Yes—Prostate samples	TIMP-3 gene promoter methylation significant in high-grade prostatic intraepithelial neoplasia and benign prostatic hyperplasia.
Jung et al. [66]	1998	TIMP-1 *	Yes—Prostate samples	TIMP-1 expression lower in PrCa versus normal tissue.
Jung et al. [68]	1997	TIMP-1 *	Yes—Prostate samples	TIMP-1 highly expressed in PrCa patients with metastasis compared to benign prostatic hyperplasia. TIMP-1 correlated with PrCa staging not grade.
Kamińska et al. [188]	2019	TIMP-2	Yes—Prostate cell lines	TIMP-2 promoter hypermethylation resulting in decreased expression in PrCa.
Karan et al. [189]	2003	TIMP-3 *	Yes—Prostate samples and prostate cell lines	TIMP-3 not expressed in PrCa cell lines, only in benign prostatic hyperplasia.
Kim et al. [179]	2012	TIMP-1 *	Yes—Prostate samples	TIMP-1 expression downregulated in metastatic PrCa.
Kuefer et al. [190]	2006	TIMP-2 *	Yes—Prostate samples and prostate cell lines	TIMP-2 over-expressed in PrCa tissue.
Kwabi-Addo et al. [191]	2010	TIMP-3	Yes—Patient samples	TIMP-3 promoter more highly methylated in PrCa versus controls.
Lee et al. [192]	2012	TIMP-2	Yes—Prostate cell lines and mice	TIMP-2 administration decreased tumor growth.
Lein et al. [79]	1999	TIMP-1 *	Yes—Prostate samples	TIMP-1 plasma concentration significantly higher in PrCa and correlated with tumor stage.
Leshner et al. [80]	2016	TIMP-2 and 3 *	Yes—Prostate samples	TIMP-2 and 3 genes do not reposition during PrCa progression.
Lichtinghagen et al. [82]	2002	TIMP-1 *	Yes—Prostate samples	TIMP-1 protein, but not mRNA, decreased in PrCa tissue.
Lichtinghagen et al. [83]	2003	TIMP-1, 2 and 3 *	Yes—Prostate samples	Expression of TIMP-2 and 3 (but not TIMP-1) decreased in PrCa tissue, with TIMP-2 correlating with stage.
Liu et al. [177]	2005	TIMP-1	Yes—Prostate samples	TIMP-1 protein levels decreased in PrCa samples, being located in secretory cells.
Lokeshwar et al. [86]	1993	TIMP *	Yes—Prostate samples	TIMP expression high in normal, but not neoplastic prostate.
Morgia et al. [95]	2005	TIMP-1 *	Yes—Prostate samples	TIMP-1 expression reduced in patients with metastatic PrCa.
Oh et al. [193]	2011	TIMP-1	Yes—Prostate samples	Elevated plasma TIMP-1 correlated with decreased survival in metastatic PrCa.
Ozden et al. [105]	2013	TIMP-1 *	Yes—Prostate samples	TIMP-2 expression in normal glands associated with lower Gleason grade.
Pulukuri et al. [194]	2007	TIMP-2	Yes—Prostate samples and prostate cell lines	Re-expression of TIMP-2 reduced tumor invasion.
Reis et al. [112]	2012	TIMP-2 *	Yes—Prostate samples	TIMP-2 under expressed in PrCa samples, but expression increased in higher grades.
Reis et al. [113]	2015	TIMP-1 and 2 *	Yes—Prostate samples	Reduced TIMP-1 expression associated with recurrence, whereas TIMP-2 expression negative in all cases.
Reis et al. [114]	2011	TIMP-1 *	Yes—Prostate samples	TIMP-1 under-expressed in PrCa samples but over-expressed in benign samples.
Riddick et al. [115]	2005	TIMP-3 and 4 *	Yes—Prostate samples	TIMP-3 and 4 expression negatively correlated with Gleason score.
Ross et al. [116]	2003	TIMP-2 *	Yes—Prostate samples	TIMP-2 expression correlated with advanced PrCa.
Ross et al. [195]	2012	TIMP-1	Yes—Prostate samples	TIMP-1 expression in blood cells upregulated in PrCa.
Sehgal et al. [123]	2003	TIMP-1 *	Yes—Prostate cell lines	TIMP-1 expression reduced in metastatic PrCa subline.
Shinojima et al. [196]	2012	TIMP-3	Yes—Prostate samples	TIMP-3 expression down regulated in PrCa versus normal due to promoter hypermethylation.
Srivastava et al. [131]	2012	TIMP-2 *	Yes—Prostate specimens	TIMP-2 GC polymorphism associated with PrCa progression not initiation, as well as cancer risk.
Stearns et al. [180]	1995	TIMP-1 *	Yes—Prostate cell lines	TIMP-1 expressed in PrCa cells.
Still et al. [134]	2000	TIMP-2 *	Yes—prostate specimens	MMP-2/TIMP-2 ratio increased in tumors of higher grade and stage.
Trudel et al. [136]	2008	TIMP-2 *	Yes—Prostate specimens	Higher TIMP-2 expression associated with longer disease-free survival.
Wood et al. [146]	1997	TIMP-1 and 2 *	Yes—Prostate samples	TIMP-1 and 2 expressed in stromal inversely correlated with Gleason score, with reduced expression in metastatic PrCa samples.
Yamanaka et al. [197]	2003	TIMP-3	Yes—Prostate samples	TIMP-3 promoter methylation low, and unchanged between PrCa and benign samples.
Yaykaşli et al. [149]	2014	TIMP-2 *	Yes—Prostate samples	TIMP-2 polymorphism under-represented in PrCa patients.
Zhang et al. [151]	2002	TIMP-1 and 2 *	Yes—Prostate samples and prostate cell lines	TIMP-1 and 2 expressed in both stromal and epithelial cells in PrCa, with no difference between fibroblasts and smooth muscle cells. Tendency for higher TIMP-2 expression in cells derived from malignant PrCa tissue.
Zhang et al. [198]	2010	TIMP-3	Yes—Prostate samples, Prostate cell lines and mice	Enforced TIMP-3 expression inhibited proliferation, survival, migration, invasion, and adhesion of cells, with reduced incidence and size of tumors in mice.

* Included in at least one other table.

**Table 4:** Studies on A Disintegrin and Metalloproteinases (ADAMs) in PrCa.

Authors	Year	ADAM	PrCa Platform	Role
Arima et al. [204]	2007	ADAM-10	Yes—Prostate cell lines and prostate samples	ADAM-10 nuclear localization significantly increased in PrCa compared to benign and correlated with Gleason score. Ablation of ADAM-10 decreased cell proliferation.
Bilgin Doğru et al. [205]	2014	ADAM-12	Yes—Prostate samples/urine	Serum and urine ADAM-12 levels significantly higher in PrCa patients compared to healthy controls, but no correlation with stage.
Burdelski et al. [199]	2017	ADAM-15	Yes—Prostate samples	ADAM-15 expression correlated to stage, Gleason grade, lymph node metastasis and PSA recurrence.
Fritzsche et al. [206]	2006	ADAM-8	Yes—Prostate samples	ADAM-8 expression correlated with higher Gleason score, but not PSA relapse-free survival.
Fritzsche et al. [207]	2008	ADAM-9	Yes—Prostate samples	ADAM-9 expression significantly higher in PrCa compared to normal tissue, and associated with shortened PSA relapse-free survival, especially in androgen-ablated patients.
Hoyne et al. [208]	2016	ADAM-19	Yes—Prostate samples and prostate cell lines	ADAM-19 expression decreased in PrCa compared to normal tissue, and positively correlated with lower grade and reduced relapse. Over-expression of ADAM-19 reduced proliferation and migration, but increased cell death.
Josson et al. [209]	2011	ADAM-9	Yes—cells	Ablation of ADAM-9 increased apoptosis, increased sensitivity to radiation and chemotherapy, and induced epithelial phenotype.
Karan et al. [189]	2003	ADAM-9, 10 and 17 *	Yes—cells and patients	Expression of ADAM-17 (but not ADAM-9 or ADAM-10) increased in PrCa compared to benign samples.
Kuefer et al. [190]	2006	ADAM-15 *	Yes—Prostate samples and prostate cell lines	Expression of ADAM-15 significantly higher in PrCa and associated with increased Gleason score and angioinvasion.
Lin et al. [201]	2012	ADAM-17	Yes—Prostate cell lines	Enforced ADAM-17 expression increased cell proliferation.
Lin et al. [210]	2012	ADAM-9	Yes—Prostate samples	ADAM-9 expression reduced in castrate-resistant PrCa compared to hormone-sensitive PrCa, with low expression in castrate-resistant PrCa associated with shorter overall survival.
Liu et al. [211]	2013	ADAM-9	Yes—Prostate cell lines and mice	Ablation of ADAM-9 decreased proliferation in vivo and tumor growth in mice.
McCulloch et al. [212]	2000	ADAM-9, 10, 11, 15 and 17	Yes—Prostate cell lines	ADAM-9, 10, 11, 15 and 17 expressed in PrCa cell lines, with androgens increasing expression of ADAM-9 and 10 while ADAM-17 was downregulated.
McCulloch et al. [213]	2004	ADAM-10	Yes—Prostate samples and prostate cell lines	ADAM-10 localized to secretory cells in PrCa with additional basal cell in benign glands.
Najy et al. [200]	2008	ADAM-15	Yes—Cells	Ablation of ADAM-15 reduced migration and adhesion in vitro and decreased bone metastasis in mice.
Peduto et al. [214]	2006	ADAM-12	Yes—Mice	ADAM-12 expressed in stromal cells adjacent to epithelial cells in PrCa. ADAM12 knock-out mice showed delayed tumor progression.
Peduto et al. [215]	2005	ADAM-9	Yes—Mice	ADAM-9 expression elevated in mouse PrCa model. ADAM-9 knock-out resulted in well differentiated tumors. Overexpression of ADAM-9 led to epithelial hyperplasia and intraepithelial neoplasia.
Pen et al. [216]	2012	ADAM-9	Yes—Prostate samples, prostate cell lines and mice	ADAM-9 nuclear expression observed in hormone refractory PrCa and in relapse patients, with levels correlated with the risk of relapse.
Rudnicka et al. [203]	2016	ADAM-28	Yes—Prostate samples and prostate cell lines	ADAM-28 expression increased in PrCa samples compared to normal tissue and in PrCa cell lines. Over-expression of ADAM-28 stimulated proliferation and migration, whereas ablation of expression or activity reduced these phenotypes.
Shigemura et al. [217]	2007	ADAM-9	Yes—Prostate cell lines	ADAM-9 expressed in AR-positive PrCa cells.
Sung et al. [218]	2006	ADAM-9	Yes—Prostate samples and prostate cell lines	ADAM-9 expression elevated in in malignant compared to benign prostate tissue. ADAM-9 expression correlated to transition to androgen-independence and cellular stress.
Xiao et al. [202]	2012	ADAM-17	Yes—Prostate cell lines	ADAM-17 expression correlated with invasiveness. Enforced expression of ADAM-17 increased invasiveness, whereas ablation decreased invasiveness.

* Included in at least one other table.

**Table 5:** Studies on A Disintegrin and Metalloproteinase with Thrombospondin Motifs (ADAMTSs) in PrCa.

Authors	Year	ADAMTS	PrCa Platform	Role
Binder et al. [221]	2020	ADAMTS-15	Yes—Prostate samples, prostate cell lines and mice	ADAMTS-15 expressed and active in PrCa samples. Enforced ADAMTS-15 expression decreased migration and proliferation but increased survival in vitro and suppressed tumor growth in mice.
Cross et al. [222]	2005	ADAMTS-1, 4, 5, 9 and 15	Yes—Prostate cell lines	ADAMTS-1, ADAMTS-4, ADAMTS-5, ADAMTS-9 and ADAMTS-15 expressed in PrCa.
Gustavsson et al. [185]	2008	ADAMTS-1 *	Yes—Prostate cell lines and mice	ADAMTS-1 expression decreased in PrCa cell line with enhanced angiogenic and tumorigenic properties, compared to parent.
Gustavsson et al. [220]	2009	ADAMTS-1	Yes—Prostate samples	ADAMTS-1 expression decreased in prostate cancer cells compared to benign prostate glands. No correlation with Gleason score, but expression lower in patients with metastatic disease.
Gustavsson et al. [219]	2010	ADAMTS-1	Yes—Prostate cell lines and mice	ADAMTS-1 ablation decreased tumor growth, but in other PrCa cells enforced expression inhibited tumor growth.
Jennbacken et al. [65]	2009	ADAMTS-1	Yes—Prostate cell lines and mice	ADAMTS-1 expression increased in slow growing tumors in mice.
Kim et al. [179]	2012	ADAMTS-1 *	Yes—Prostate samples	ADAMTS-1 mRNA overexpressed in PrCa samples.
Molokwu et al. [223]	2010	ADAMTS-1 and 15	Yes—Prostate cell lines	ADAMTS-15 (but not ADAMTS-1) expressed in PrCa.

* Included in at least one other table.

**Table 6:** Search terms and inclusion/exclusion criteria used in this systematic-like review.

	Inclusion Criteria	Exclusion Criteria
Population	Men, mice/rats or cells.	Involved other animal species or cells not related to PrCa progression.
Concept	All human metzincin superfamily members, including members of the MMP, MT-MMP, ADAM, ADAMTS, BMP1/TLL and Meprin subgroups, as well as the TIMP family of regulators.	Examining proteins other than metzincin superfamily members or TIMPs, or in different cancer types, other diseases, or normal biology.
Context	Studies that investigated the role of the metzincin superfamily in PrCa progression.	Did not specially look at the role of metzincin superfamily members in PrCa cancer progression.
